# Bee Pollen Extracts: Chemical Composition, Antioxidant Properties, and Effect on the Growth of Selected Probiotic and Pathogenic Bacteria

**DOI:** 10.3390/antiox11050959

**Published:** 2022-05-12

**Authors:** Cornelia-Ioana Ilie, Eliza Oprea, Elisabeta-Irina Geana, Angela Spoiala, Mihaela Buleandra, Gratiela Gradisteanu Pircalabioru, Irinel Adriana Badea, Denisa Ficai, Ecaterina Andronescu, Anton Ficai, Lia-Mara Ditu

**Affiliations:** 1 Department of Science and Engineering of Oxide Materials and Nanomaterials, Faculty of Chemical Engineering and Biotechnologies, University Politehnica of Bucharest, 1–7 Gh. Polizu Street, 011061 Bucharest, Romania; cornelia_ioana.ilie@upb.ro (C.-I.I.); angela.spoiala@upb.ro (A.S.); ecaterina.andronescu@upb.ro (E.A.); 2 National Centre for Micro and Nanomaterials and National Centre for Food Safety, Faculty of Chemical Engineering and Biotechnologies, University Politehnica of Bucharest, 313 Splaiul Independentei, 060042 Bucharest, Romania; denisa.ficai@upb.ro; 3 Department of Microbiology and Immunology, Faculty of Biology, University of Bucharest, 1–3 Aleea Portocalelor, 060101 Bucharest, Romania; lia-mara.ditu@bio.unibuc.ro; 4 Department of Organic Chemistry, Biochemistry and Catalysis, Faculty of Chemistry, University of Bucharest, 030018 Bucharest, Romania; 5 National R&D Institute for Cryogenics and Isotopic Technologies—ICIT, 4th Uzinei Street, 240050 Râmnicu Vâlcea, Romania; irina.geana@icsi.ro; 6 Department of Analytical Chemistry, Faculty of Chemistry, University of Bucharest, 90–92 Șoseaua Panduri, 050663 Bucharest, Romania; mihaela.buleandra@g.unibuc.ro (M.B.); irinel.badea@chimie.unibuc.ro (I.A.B.); 7 Research Institute of the University of Bucharest, 050095 Bucharest, Romania; gratiela.gradisteanu@icub.unibuc.ro; 8 Department of Inorganic Chemistry, Physical Chemistry and Electrochemistry, Faculty of Chemical Engineering and Biotechnologies, University Politehnica of Bucharest, 1–7 Gh. Polizu Street, 011061 Bucharest, Romania; 9 Academy of Romanian Scientists, 3 Ilfov Street, 050045 Bucharest, Romania

**Keywords:** bee pollen, phenolic compounds, antimicrobial activity, prebiotics, tumour proliferation inhibitory agents

## Abstract

This paper evaluated the chemical and biological properties of bee pollen samples from Romania. Firstly, the bee pollen alcoholic extracts (BPEs) were obtained from raw bee pollen harvested by *Apis mellifera carpatica* bees. The chemical composition of BPE was obtained by determination of total phenol content and total flavonoid content, UHPLC-DAD-ESI/MS analysis of phenolic compounds, and GC-MS analysis of fatty acids, esters, and terpenes. Additionally, the antioxidant activity was evaluated by the Trolox Equivalent Antioxidant Capacity method. Furthermore, the biological properties of BPE were evaluated (antimicrobial and cytotoxic activity). The raw BP samples studied in this paper had significant phenolic acid and flavonoid content, and moderate fatty acid, ester, and terpene content. P1, P2, and P4 have the highest TPC and TFC levels, and the best antioxidant activity. All BPEs studied had antimicrobial activity on pathogenic strains isolated from the clinic or standard strains. A synergistic antimicrobial effect of the BPEs was observed along with the soluble compounds of *L. rhamnosus* MF9 and *E. faecalis* 2M17 against some pathogenic (clinical) strains and, considering the tumour proliferation inhibitory activity, makes BP a potential prebiotic and antitumour agent for the gut environment.

## 1. Introduction

Working bees produce bee pollen from flower pollen, nectar, or honey, adding salivary substances. It is stored in the beehive and used as a food source for the whole colony [1]. It can be classified by water content in raw (fresh) bee pollen, which contains 21–30% water and dry pollen obtained from raw pollen by drying at a temperature up to 45 °C, with final water content between 2–9% [2].

The morphological and structural analysis of pollen grains under the microscope allows for the identification of species, family, or genus from which they originate and determines the percentage of each type of pollen, and its respective classification (monofloral or multifloral) [3]. When the product contains more than 90% pollen from a single species, it is considered monofloral, and its name is specified [1,2,4,5]. There are no standards or regulations at the European Union level or norms imposed by other agencies regarding this product’s quality, physical-chemical properties, and microbial load [6]. Additionally, the Food and Drug Administration (FDA) does not recognise bee pollen (BP) as a food supplement. It is therefore not marketed worldwide according to standards [6]. However, some states have imposed quality standards on physical–chemical properties, with no restrictions on the pesticide content or the microbial load of this bee product [3,6,7].

The chemical composition of the BP depends on the plant, the geographical source, the climatic conditions, the type of soil, and the species of bees that harvest [1,8]. As a result, pollen is a very varied plant product, rich in biologically active substances. About 250 substances have been identified from different plant species [1]. The main chemicals that could be present are amino acids, proteins, carbohydrates (fructose, glucose, sucrose, maltose, trehalose, etc.), lipids (fatty acids-FA, phospholipids, sphingolipids, triglycerides), nucleic acids, phenolic compounds, mineral elements (aluminium, calcium, copper, chromium, iron, etc.), enzymes and coenzymes, carotenoids, volatile oils as well as vitamins (group-B, C, D, E, H, K) [1,3,4,9,10,11].

Bee pollen can be used as an adjuvant, along with drugs (for example, chemotherapeutics), in treating several pathologies due to its medical potential and nutritional applications [1,12,13]. In addition, BP seems to become an increasingly appreciated functional food either administered or added to other foods, both for human use and supplementing animal feed (for meat for human consumption). In the first case, there is an increasing trend towards being used daily by humans for a series of effects, such as stimulating blood circulation, increasing immunity, enhancing physical and mental activities, etc. On the other hand, adding to other foods improves those foods’ nutritional, functional, and sensory properties [14,15]. Moreover, BP is considered a superfood and an essential source of compounds with antimicrobial activity, such as phenolic acids, flavonoids, FA, sterols, and alkaloids [16]. The metabolites of microbial cells from the BP microbiome seem to have a contribution as well [17]. 

The bee pollen’s antimicrobial activity (and chemical composition, obviously) strongly depends on botanical origin, the type of solvent used for extraction, storage conditions, etc. The major published papers have shown that Gram-negative bacteria are, in general, less sensitive to bee pollen extracts (BPEs) than Gram-positive ones [18,19,20,21,22,23,24], excluding only a few cases [25,26,27]. 

Some researchers postulated that phenolic compounds from BP have stimulating effects on the growth of beneficial bacteria from gut microbiota and, at the same time, inhibit pathogenic bacteria [28,29]. 

This paper presents the composition of phenolic compounds by chromatography of five samples of raw pollen purchased directly from beekeepers, and quantitative assessments based on the spectrophotometric method (total phenols and total flavonoids). In addition, antioxidant capacity, antimicrobial activity, and cytotoxic activity on the tumoural cell line were also evaluated. The first has aimed at qualitative and quantitative analysis, a semiquantitative assessment of adherence to the inert substrate, and the antimicrobial effect of bee pollen on the adherence capacity of microbial strains with pathogenic potential. Additionally, the prebiotic effect of the bee pollen on the adherence capacity and the growth curve of microbial strains with probiotic potential were evaluated. Furthermore, the novelty of this study is the assessment of the antimicrobial effect of the bee pollen and soluble compounds of lactic strains (at the same time) on the adherence capacity to sensitive cellular substrates of some pathogenic strains. 

## 2. Materials and Methods

### 2.1. Materials

The bee pollen samples were provided by beekeepers from Romania in a pollution-free area (Chiojdeanca, Prahova District) and were stored at −45 °C in tightly closed bottles. The samples came from pollen harvested by bees (*Apis mellifera carpatica*) in 2020, from plant species of spontaneous flora throughout the spring (Table 1), at different time intervals depending on climatic conditions and the diversity of flowering plants. 

### 2.2. Reagents

The determination of total phenol content (TPC), total flavonoid content (TFC), and Trolox Equivalent Antioxidant Capacity (TEAC) was performed using Folin–Ciocâlteu’s phenol reagent, gallic acid by Merck (Darmstadt, Germany). Ethanol, sodium carbonate, aluminium chloride; 2,2′-azino-bis (3–ethylbenzothiazoline-6-sulfonate), and potassium persulfate were purchased from Sigma-Aldrich (Darmstadt, Germany).

All reagents for ultra-high-performance liquid chromatography diode array detector electrospray ionisation tandem mass spectrometry (UHPLC-DAD-ESI/MS) presented high analytical purity. Merck supplied methanol and formic acid, and phenolic standards were from Sigma-Aldrich. Deionised water, produced by a Milli-Q Millipore system (Bedford, MA, USA), was used to prepare aqueous solutions and UHPLC mobile phases. Syringe filters (13 mm, polytetrafluoroethylene membrane 0.45 μm) were purchased from Supelco (Darmstadt, Germany).

GC-MS analysis was performed using alkane standard solutions and hexane (chromatographic purity) from Sigma-Aldrich.

The observation of microbiological activity was performed using Nutrient Broth No. 2 (NB), Sabouraud Glucose Agar with Chloramphenicol, Yeast Peptone Glucose Extract Agar (YPG), Mueller–Hinton Broth (M-H), Man–Rogosa–Sharpe Broth (MRS), agar, methanol, ethanol, acetic acid, crystal violet/hexamethylpararosaniline chloride and Giemsa stain acquired from Sigma-Aldrich. All strains tested in this study were obtained from the Microorganisms Collection of the Department of Microbiology, Faculty of Biology & Research Institute of the University of Bucharest. Assessing the degree of cell adherence was performed using Eagle MEM (EMEM), foetal bovine serum (FBS), unessential amino acids, and gentamicin, which were purchased from Gibco BRL (Boston, MA, USA). The cytotoxicity assays were performed using 3-[4,5-dimethylthiazole-2-yl]-2,5-diphenyltetrazolium bromide (MTT) and lactate dehydrogenase (LDH) kits, which were acquired from Roche (Basel, Switzerland). Hep-2 CCL-23^TM^ were provided by ATCC (Manassas, VA, USA). The cells were cultured in Dulbecco’s Modified Eagle’s medium DMEM (Thermo Fischer Scientific, San Jose, CA, USA), FBS, penicillin and streptomycin (Gibco).

### 2.3. Bee Pollen Extract (BPE) Preparation

The extractions were performed by heating 2 g fresh bee pollen at 40 °C with ethanol 70% (*v*/*v*) using an ultrasonic bath (Elmasonic S80H, Singen, Germany) with high-performance 37 kHz sandwich systems, making possible the extraction of active compounds by causing cell lysis [30,31]. This method contains more steps, such as vortexing, ultrasonication, and centrifugation. The final volume of BPE was 50 mL for all samples, and they were stored in tightly closed bottles at −45 °C. 

### 2.4. Chemical Composition of BPE

#### 2.4.1. Determination of Moisture

The loss from drying was determined by drying 1–2 g of the BP sample at 105 °C in weighing vials to constant mass [32,33,34]. The Memmert UF55 oven (Schwabach, Germany) and the analytical balance (RADWAG AS 160.R2, Torunska, Poland) were used. The experiment was performed in triplicate. 

#### 2.4.2. Determination of Total Phenol Content (TPC)

Determination of total phenol content was done using the Folin–Ciocâlteu method [35]. The principle of the method is based on the reduction of phosphomolybdic and phosphotungstic acids from the Folin–Ciocâlteu reagent by phenolic compounds of BPE. The reaction occurs in a basic medium, resulting in Mo^4+^ ions (blue), which allow spectrophotometer analysis of absorbance at 745–750 nm. This determination was assayed by homogenising a 0.5 mL sample or standard (gallic acid) in a 5 mL Folin–Ciocâlteu reagent and adding 4 mL of 1 M sodium carbonate. Absorbance was measured after 15 min and compared to a blank sample (containing ethanol 70% *v*/*v*) in 1 cm glass cuvettes. The absorbance of samples was determined at 746 nm with a Shimadzu UV-1800 spectrophotometer (Kyoto, Japan). A calibration curve was plotted with standard solutions of gallic acid with concentrations varying between 5 and 150 mg/L. TPC was expressed as milligram (mg) of gallic acid equivalent (GAE)/gram (g) of the sample [36]. The experiment was performed in triplicate.

#### 2.4.3. Determination of Total Flavonoid Content (TFC)

The total flavonoid content of the BPE was determined by the aluminium chloride method [36]. This method supposes filtering BPE after adding 10 mL solution of sample or standard of different concentrations and 10 mL of 10% sodium acetate. The filtered solution (5 mL) was added to 3 mL of 2.5% AlCl_3_ solution and filled with ethanol in a 25 mL volumetric flask. After stirring, this solution was incubated in the dark and at room temperature for 45 min. The absorbance of samples was measured at 430 nm using 1 cm glass cuvettes. The analytic standard for the calibration curve was quercetin of different concentrations. The TFC was expressed in mg quercetin (QE)/g BP; it was calculated using the calibration curve obtained for concentrations of quercetin varying between 0 and 0.12 mg/mL [36]. The experiment was performed in triplicate.

#### 2.4.4. Phenolic Compound Analysis by UHPLC-DAD-ESI/MS 

The measurements were performed using an UltiMate 3000 UHPLC system (ThermoFisher Scientific, Darmstadt, Germany) consisting of a quaternary pump; DAD set at 280 nm, column oven and autosampler coupled to a Q Exactive™ Focus Hybrid Quadrupole-OrbiTrap mass spectrometer equipped with heated electrospray ionisation (HESI) (ThermoFisher Scientific). Chromatographic and mass spectrometric parameters have been set according to Ciucure and Geană [37].

Phenolic acids and flavonoids from BPE were identified and quantified according to mass spectra, accurate mass, and characteristic retention time against external standard solutions analysed under the same conditions, covering a calibration range between 0 and 10 mg/L [37]. Data-dependent scans with collision-induced dissociation (CID) set between 15 and 60 eV were performed for fragmentation studies to confirm each analysed phenolic compound. The Xcalibur software (Version 4.1) was used for instrument control, data acquisition, and data analysis. In contrast, the ChemSpider internet database of accurate MS data (www.chemspider.com; accessed on 1 September 2020) was used as a reference library to identify compounds of interest.

#### 2.4.5. GC-MS Analysis

GC-MS analysis was carried out using a Thermo Fisher system (Focus GC and Polaris Q ion trap mass detector) equipped with a TriPlus autosampler and Xcalibur^®^ software. The chromatographic separation was performed on a DB-5MS capillary column (25 m × 0.25 mm; 0.25 μm of film thickness), and helium 6.0 was used as the carrier gas (1 mL/min). The oven temperature program began at 60 °C and was held for 3 min, ramped to 200 °C at a rate of 10 °C/min, and then ramped to 240 °C at a rate of 12 °C/min and held for 2 min. The temperatures of the MS transfer line and the ion source were 250 and 200 °C, respectively. The BPE samples were treated with hexane (1:1, *v*/*v*) after the ethanol was evaporated. The mixture obtained was vortexed, centrifuged, and filtered with PTFE membrane (0.45 μm) [38,39,40]. In splitless mode, a volume of 1 µL of the liquid sample was injected into the GC inlet port with the temperature maintained at 250 °C. The detector operated in positive ionisation (70 eV) in full-scan mode from 40 to 500 *m*/*z*. 

The percentage composition of the identified compounds was calculated from the total ion chromatogram based on GC peak areas. Compound identification was made according to their retention indices (RI) and mass spectra found in databases (Wiley, NIST) and the literature [41]. The RIs were calculated using the retention times of C_8_–C_20_ n-alkanes injected in the same chromatographic conditions [42].

### 2.5. Determination of Trolox Equivalent Antioxidant Capacity (TEAC)

This method was realised by highlighting the neutralisation potential of the cation radical ABTS^•^^+^ (2,2′-azino-bis (3–ethylbenzothiazoline-6-sulfonate), expressed as Trolox equivalents. 

The radical ABTS^•^^+^ is an unphysiological radical. From a thermodynamic point of view, a compound can react with ABTS^•^^+^ if it has less redox potential than this radical (0.68 V). Most phenolic compounds have a lower redox potential than ABTS, so they can react with it. Using a short, fixed time of determination (usually 4 or 6 min) cannot ensure the complete reaction of all active compounds, thus underestimating the total antioxidant capacity of the sample.

The TEAC method applied is spectrophotometric and highlights the capacity of the tested compound to discolour the radical ABTS^•^^+^ (blue-green chromophore). The radical ABTS^•^^+^ has maximum absorption at 415, 645, 734, and 815 nm. Still, the wavelength 734 nm is most used for monitoring the reaction.

A stable stock solution of ABTS^•^^+^ was made by reacting an aqueous solution of ABTS with potassium persulfate and left in the dark at room temperature for 12–16 h before use. Then the solution was diluted with ethanol (to an absorbance of around 0.7) to obtain the working solution of ABTS^•^^+^ [43,44]. The results obtained from the samples were expressed in millimoles of Trolox equivalent (mmol Trolox)/g BP. The experiment was performed in triplicate.

### 2.6. Methods Applied in the Biological Activity of BPE

#### 2.6.1. Qualitative Evaluation of Antimicrobial Activity

The antimicrobial activity was performed using the following standard strains: *Enterococcus faecalis* ATCC 19433, *Staphylococcus aureus* ATCC 25422, *Escherichia coli* ATCC 25923, *Pseudomonas aeruginosa* ATCC 25785, *Candida albicans* ATCC 1688; and clinically isolated strains: *Enterobacter cloacae*, *Salmonella* spp., *Candida famata*, *Candida glabrata*, *Candida guillermondii*, *Candida krusei*, and *Candida lusitaniae*. 

The antimicrobial activity was evaluated using an adapted spot diffusion method [45,46,47]. Bacterial cell suspensions of 1.5 × 10^8^ CFU/mL (0.5 McFarland density standard) and yeast suspensions of 3 × 10^8^ CFU/mL (1 McFarland density standard) were obtained from cultures on Nutrient Agar medium (for bacterial species) and Sabouraud Agar medium (for yeast species). Petri dishes with Nutrient Agar or Sabouraud Agar were seeded with inoculums, and 20 µL of each solution of BPE was spotted. Due to the BPE samples containing ethanol, a control (C_Et_) was performed. The negative control was considered the sterile medium, and the positive control (C+) was the broth medium inoculated with microbial suspensions. After diffusion, the plates were incubated at 37 °C for 24 h for bacterial strains and 48 h for yeast strains. 

#### 2.6.2. Quantitative Evaluation of Antimicrobial Activity

Determination of minimum inhibitory concentration (MIC) was performed by an adapted binary serial microdilution standard method [46,47] in liquid medium (NB for bacteria and YPG for yeasts), using 96-well microtiter plates. From each BPE sample, serial two-fold microdilutions were achieved in 150 μL of corresponding broth medium seeded with the standard inoculum of 1.5 × 10^8^ CFU/mL (for bacterial strains) and 3 × 10^8^ CFU/mL (for yeast strains). Due to the BPE samples containing ethanol, a control (C_Et_) was performed. The negative control was considered the sterile liquid medium, and the positive control (C+) was the broth medium inoculated with microbial suspensions, following the same conditions described before. The dishes were incubated at 37 °C for 24 h for bacterial strains and 48 h for yeast strains. The MIC values were established by visual analysis and spectrophotometric measuring of absorbance at 620 nm using a BIOTEK SYNERGY-HTX ELISA multi-mode reader (Winooski, VT, USA). 

#### 2.6.3. Semiquantitative Assessment of Microbial Adherence to the Inert Substratum 

The microtiter broth method was performed to evaluate biofilm development on the inert substrate using the same serial two-fold microdilution method. After 24 h of incubation, the liquid medium from 96-well plates (containing binary dilution of the samples tested) was discarded, the wells were washed three times with sterile physiological buffer saline (PBS), and the bacterial cells adhered to the walls were fixed with cold methanol for 5 min, followed by stain with 1% crystal violet solution for 15 min. The dyed biofilm was resuspended by 33% acetic acid solution, and the absorbance of the blue solution was measured at 490 nm using a BIOTEK SYNERGY-HTX ELISA multi-mode reader [46,47]. 

#### 2.6.4. Evaluation of the Inhibitory Effect of the BPE Samples on the Ability of the Tested Microbial Strains to Attach to the Cellular Substrate 

The microbial adherence test was performed using a Hep-2 (*human epithelioma*) cell line, inoculated on 6-well cell culture plates in a final density of 1 × 10^5^ cells/well EMEM culture medium supplemented with 10% FBS, 0.1 mM unessential amino acids solution, 0.5 mL (50 µg/mL) gentamicin, and followed by incubation for 24 h at 37 °C in a humid atmosphere with 5% CO_2_. When the cells reached 80–100% confluence, the plates were used in the experiment.

The ethanol from BPE samples was evaporated at 30 °C and resuspended in ultrapure water (10 mL BPE: 1 mL ultrapure water). 

The following microbial strains were used: *E. faecalis* ATCC 19433, *S. aureus* ATCC 25422, *E. cloacae*, *P. aeruginosa* ATCC 25785, *C. albicans* ATCC 1688, *C. famata*, *C. glabrata*, *C. guillermondii*, *C. krusei*, and *C. lusitaniae*. The Hep-2 cell monolayer was inoculated with 100 µL BPE, 1 mL microbial suspensions in PBS, with a standard density of 1.5 × 10^8^ CFU/mL (0.5 McFarland density) for bacterial and 3 × 10^8^ CFU/mL (1 McFarland density) for yeast strains. The dishes were incubated at 37 °C for 1.5 h in a CO_2_ atmosphere. After incubation, the plates were washed three times with PBS, fixed with cold methanol (4 °C) for 5 min and stained with 10% Giemsa solution for 15–20 min. The wells were washed three times with water, dried at room temperature, and examined via Zeiss Primo Star microscope (Oberkochen, Germany) to determine the adherence pattern and the adherence index (AI%) calculated as the ratio between the number of Hep-2 cells with adherent bacteria and the total number of Hep-2 cells [48,49,50].

#### 2.6.5. Evaluation of the Prebiotic Effect of the BPE Samples on the Ability of Two Microbial Strains with Probiotic Potential to Adhere to a Cellular Substrate

Two probiotic strains isolated from newborn faeces were used in the experiment: *Lactobacillus rhamnosus* MF9 and *Enterococcus faecalis* 2M17. The working protocol was identical to the one described above, indicating that two working models were used in the experiment: BPE: bacterial suspension = 1:10 and 2:10, respectively (adapted method [51]).

#### 2.6.6. Assessment of the Prebiotic Effect of the BPE on the Growth Curve for Two Microbial Strains with Probiotic Potential

Determination of growth curves for *L. rhamnosus* MF9 and *E. faecalis* 2M17 was performed by following their growth and multiplication under the influence of BPE for 26 h. Thus, in the 96-well plate, 20 μL of bacterial suspension was inoculated in 200 μL of broth medium specific to each strain (MRS for *L. rhamnosus* MF9, M-H for *E. faecalis* 2M17) (volumetric ratio between the volume of inoculated microbial suspension and that of the broth medium = 1:10), followed by the addition of 20μL of extract and 40 μL of extract, respectively. The plates were incubated at 37 °C in anaerobic conditions, and the growth was evaluated at different times of incubation, starting with 0 min (T_0_), 2 h, 6 h, 8 h, 10 h, 12 h, 24 h, and 26 h, by spectrophotometric reading at λ = 620 nm (for the MRS medium) and λ = 600 nm (for M-H medium). Simultaneously, serial dilutions from each sample were inoculated on MRS/M-H agar plates in triplicate, and viable cell counts were assessed after incubation at 37 °C for 24 h in anaerobic conditions [49,50,51,52]. 

#### 2.6.7. Assessment of the Synergic Influence of BPE and Probiotic Soluble Compounds on the Capacity of Some Pathogenic Strains to Adhere to the Cellular Substratum

This experiment was performed using two pathogenic strains isolated from clinical samples (urine): *E. cloacae* and *C. guillermondii*. Microbial suspensions were made in PBS using fresh cultures (of 18–24 h) with a standard density of 1.5 × 10^8^ CFU/mL (0.5 McFarland density) for bacterial strains and 3 × 10^8^ CFU/mL (1 McFarland density) for yeast strains. The probiotic strain cultures obtained in MRS liquid media with Tween 80 were centrifuged for 10 min at 3500 rpm. The supernatants containing soluble compounds secreted by *L. rhamnosus* MF9 and *E. faecalis* 2M17 were separated, and the pH was adjusted to 7.2 (±2). 

The microbial adherence test was performed using a Hep-2 cell line inoculated on 6-well cell culture dishes in the conditions described in Section 2.6.4. The working scheme was as follows: in each well 50 µL BPE, 1 mL pathogenic bacterial suspension, and 100 µL probiotic supernatant fraction were added; the following controls were used: pathogenic strain adherence control (C_p_) and pathogenic strain with probiotic supernatant adherence control (C_P+SN_). After incubation, the wells were washed three times with water, dried at room temperature, and examined via Zeiss Primo Star microscope (Oberkochen, Germany) to determine the adherence pattern and the adherence index (AI%) calculated as the ratio between the number of Hep-2 cells with adherent bacteria and the total number of Hep-2 cells [49].

### 2.7. Cytototoxic Activity of BPEs

The cytotoxic activity of BPE was evaluated using the Hep-2 CCL-23^TM^ (tumour) cell line. Initially, the cells were seeded in 24-well plates with a density of 1 × 10^5^/mL and incubated for 24 h at 37 °C in a humidified atmosphere (95% air and 5% CO_2_) containing DMEM supplemented with 10% FBS and 1% penicillin-streptomycin. Cell viability and proliferation potential of the BPEs were evaluated using MTT assay after 2 and 6 days of incubation with the BPEs. Each sample was incubated with a 1 mg/mL MTT solution for 4 h at 37 °C, and the absorbance at 570 nm was measured via Flex Station 3 spectrophotometer (San Jose, CA, USA).

Further, the cytotoxic potential of all BPEs was evaluated by LDH assay, which indicates the number of dead cells in the culture. The culture media was mixed with the components of the LDH kit according to the manufacturer’s instructions and incubated in darkness for 20 min, followed by spectrophotometric measurement at 490 nm. The cell culture (without BPE) was used as a control, and the experiments were done in triplicate [53]. 

### 2.8. Statistical Analysis

The data results were statistically analysed using the GraphPad Prism 9 program for Windows 64-bit, version 9.3.1 (471), developed by GraphPad Software, (San Diego, CA, USA). All experiments were performed in three independent determinations. The results were expressed as ± SD (standard deviation), statistically analysed using one-way analysis of variance (one-way ANOVA) and Tukey’s multiple comparisons test, or repeated measures one-way ANOVA (RM one-way ANOVA) and Dunnett’s multiple comparisons test. *p*-Value < 0.05 is considered statistically significant. 

## 3. Results and Discussions

### 3.1. Chemical Composition of BPE

The chemical composition of BP has preoccupied researchers from various countries (Portugal [19], [Turkey [54], Poland [55], China [39], Algeria [40], etc.) over time and continues to be a current topic of interest.

Bee pollen has a complex composition that depends on many factors [39]. The classes of compounds of interest, in terms of antioxidant and antimicrobial activity, are mainly phenolic compounds, terpenes, and fatty acids, which will be presented below.

#### 3.1.1. Determination of Moisture

The drying loss results (mainly moisture content of fresh bee pollen) are listed in Table 2 and are expressed as a percentage (%) ±SD.

The values varied between 22.62 and 35.87% and are similar to Mărgăoan et al. [33], who analysed fresh BP samples collected from different regions in Transylvania, Romania.

#### 3.1.2. Determination of TPC, TFC, and TEAC

The TPC in the BPEs was determined using a calibration curve, the results being expressed in mg total phenols defined as gallic acid/g BP. The equation of the calibration curve was y = 0.0057x + 0.0349 and R^2^ = 0.9995.

The TFC was performed similarly. The calibration curve equation was y = 0.0624x − 0.0024 and R^2^ = 0.9946, while results were expressed as mg quercetin (QE)/g BP. 

After plotting the calibration curve with a Trolox solution, the total antioxidant capacity (TEAC) was obtained by interpolation. The curve equation was y = 17.472x − 2.8971, and R^2^ = 0.9925. The TPC, TFC, and TEAC results are presented in Table 3 as mean value ± SD.

According to the results shown in the previous table, P1, P2, and P4 have the highest TPC and TFC levels. In our study, TPC values in BPE samples vary between 10.77 ± 0.015 (P5) and 16.15 ± 0.015 mg GAE/g BP (P2). The TFC values range between 0.19 ± 0.01 (P5) and 0.30 ± 0.01 mg QE/g BP (P2). The BPE antioxidant activity data (TEAC method) correlates with TPC and TFC results. It can be observed (Table 3) that P1, P2, and P4, which have a higher content of both phenols and flavonoids, also have the best antioxidant activity which correlates with other studies [43,56]. In a previous study, Mărghitaș et al. [57] obtained similar results through applying the same method to TPC of the fresh BP samples provided from the species of *Crataegus monogyna* (15.3 mg GAE/g BP), *Taraxacum officinale* (16.2 mg GAE/g BP) and *Salix* sp. (16.4 mg GAE/g BP). Additionally, Mărgăoan et al. [58] determined the highest values of TPC from the BPE of *Rosaceae* (38.53 mg GAE/g BPE) and the lowest values (17.96 mg GAE/g BPE) for BPE from the *Fagaceae* family. The TFC ranged between 7.72 and 2.39 mg QE/g BPE. Furthermore, Alimoglu et al. [59] showed the highest TPC value (26.92 ± 0.32 mg GAE/g hydroalcoholic extract) for bee pollen provided mainly from *Salix* sp. According to this study, the TPC values are correlated with TFC results. The TPC value for BPE from *Rosaceae* (47%) is 18.80 ± 0.91 mg GAE/g hydroalcoholic extract and 8.94 ± 0.51 mg QE/g hydroalcoholic extract. Additionally, for BPE that contains approx. 48% *Taraxacum* sp., the TPC value is 17.82 ± 0.45 mg GAE/g hydroalcoholic extract and for TFC is 6.21 ± 0.75 mg QE/g hydroalcoholic extract. Leja et al. [56] mentioned that *Robinia pseudoacacia* bee pollen has very high antioxidant activity and the highest phenolic compound content compared to BP from other plant species. However, it is not confirmed in the case of the P5 sample. A possible explanation could be the unfavourable weather conditions (late frost) of the harvesting year, which affected some trees during the flowering period, including the acacia tree (which bloomed in 2020 in two stages). At the same time, it is known that temperature influences the accumulation of plant secondary metabolites, including phenolic compounds [60].

#### 3.1.3. Phenolic Compound Profile by UHPLC-DAD-ESI/MS

The results presented in Table 4 show the identification and quantification of 20 phenolic compounds analysed by UHPLC-DAD-ESI/MS in all BPE samples. Total Ion Current (TIC) chromatogram of the BPEs in the negative ion mode covered a scan range between 100–1000 *m*/*z*. The extracted chromatograms (using a 5 ppm mass accuracy window) of the main compounds identified in the BPEs are presented in Figure S1.

According to these results, each BPE sample had its phenolic profile. Some phenolic acids were present in significant quantities: 4-hydroxybenzoic acid (19.770 µg/g BP in P2), chlorogenic acid (46.939 µg/g BP in P4), and ferulic acid (2.978 µg/g BP in P1), while flavonoids were sometimes detected in larger quantities than phenolic acids. For example, P3, P4, and P1 contain 135.301 µg/g BP, 69.45 µg/g BP, and 45.662 µg/g BP rutin. On the other side, quercetin was present in smaller quantities: 7.883 µg/g BP and 5.981 µg/g BP in P4 and P5, respectively, which also contains isorhamnetin (40.220 µg/g BP) and kaempferol (26.472 µg/g BP).

These results are consistent with Bayram et al. [61], who detected, in BP samples provided from *Malus* sp. and *Prunus* sp., caffeic acid (0.4 ± 0.04 µg/g BP), *p*-coumaric acid (0.78 ± 0.01 µg/g BP), rutin (34.45 ± 1.03 µg/g BP), quercetin (6.17 ± 0.18 µg/g BP), and isorhamnetin (3.37 ± 6.75 µg/g BP);which are similar to P3 from our study. Additionally, in the cited paper, BP samples from *Rosaceae*, *Taraxacum* sp., *Salix* sp., *Pyrus* sp., and *Hypericum* sp. are identified as polyphenols similar to BPE from the present study. Gercek et al. [54] investigated BP that comes from *Asteraceae*, *Fabaceae*, *Campanulaceae*, *Cistaceae*, and *Rosaceae* family 23 polyphenols and determined high concentrations for rutin (115.442 ± 7.77 µg/g BP), quercetin (7.849 ± 5.28 µg/g BP), kaempferol (9.870 ± 0.79 µg/g BP), and isorhamnetin (0.523 ± 0.032 µg/g BP). Lower concentrations showed myricetin (2.220 ± 0.177 µg/g BP), *p*-coumaric acid (1.508 ± 0.091 µg/g BP), caffeic acid (0.928 ± 0.074 µg/g BP), gallic acid (0.585 ± 0.031 µg/g BP), syringic acid (0.284 ± 0.017 µg/g BP), and catechin (0.037 ± 0.002 µg/g BP) [54].

#### 3.1.4. BPE Analysis by GC-MS

The results of GC-MS analysis for FA, esters, and terpenes from all BPE samples are presented in Table 5 as percentages (%). GC-MS analysis reveals that three compounds were unidentified: for P1, one compound with a percentage composition of 1.41%; for P3, two compounds with a total percentage composition of 2.63%; and for P5, three compounds with a total percentage composition of 9%.

Based on the GC-MS analysis, 28 compounds were detected and quantified. Some terpenes were presented as minor constituents (less than 1% compound/ BP sample). FA is identified in higher percentages, such as stearic acid methyl ester (88.31% for P4), linolenic acid (43.42%—P5; 43.23%—P3), linoleic acid (19.40%—P1), and palmitic acid (13.53%—P2). The results confirm that the chemical profile variability of BP samples is dependent on the plant species from which it was harvested by bees [62,63,64]. As shown in Table 1, P1 and P2 came from similar plants (according to spontaneous flora at BP harvest). This is confirmed in Table 5 by the similar FA, ester, and terpene profiles. The relative samples’ composition changes significantly to the content of the FA, esters, and terpenes, and a pattern does not seem very easy to identify. However, the quantitative analysis of these compounds is worth investigating in detail due to their significant antimicrobial potential. In addition, some BP could be a valuable source of polyunsaturated fatty acids, which have been recognized as regulators of the antioxidant signalling pathway, and for their anti-inflammatory properties [65]. 

### 3.2. Biological Activity of BPE

#### 3.2.1. Qualitative Evaluation of Antimicrobial Activity

Antimicrobial activity was qualitatively evaluated by measuring the diameters of the inhibition zones that appeared around the spot (of BPE samples) and expressing them as mean values ±SD (Table 6). 

In our study, all BPEs tested have shown antimicrobial activity against pathogenic strains, and Gram-positive bacteria were more sensitive than Gram-negative bacteria and yeasts. P1 and P4 determined sensitivity to *E. faecalis* ATCC 19433, and P5 against *S. aureus* ATCC 25422. Didaras et al. [16] reported that Gram-positive bacteria are more susceptible to bee-collected pollen, although they also admit some exceptions.

The inhibitory effect toward the Gram-negative bacteria was more evident for P2, which expressed a large inhibition zone for all tested strains after excluding the C_Et_. The most sensitive Gram-negative strains were *E. coli* ATCC 25922, followed by *P. aeruginosa* ATCC 25785. The inhibitory activities of plant flavonoid compounds (contained in P2) against Gram-negative bacteria were less reported. A scientific explanation of their mechanism of action is hard to draw. 

It is known that Gram-negative bacteria, generally with higher natural resistance to antibiotics (ATBs), have a cell wall composed of two major components: the peptidoglycan-lipoprotein complex and the outer membrane that functions as an additional barrier to permeability [19,66]. Velásquez et al. [67] and Santa Barbara et al. [66] have reported that BPEs tested presented antimicrobial activity on a Gram-negative strain (*P. aeruginosa* ATCC 15442). At the same time, the last paper mentions that BP had a similar effect, including on two other isolated micro-organisms from biological fluids (from the clinic) belonging to the same species.

The antifungal activity of tested samples was evaluated on six yeast strains (one *C. albicans* standard strain and five different clinical strains). The most sensitive strain was *C. krusei*, with the highest area of inhibition expressed against the P4 sample (Table 6). The results presented in Table 4 show that P4 has a high chlorogenic acid content (46.939 µg/g). According to different research regarding antifungal activity, the chlorogenic acid breaks down the membrane permeability barrier, generating pores in the cell membrane and causing the leakage of ions and other materials [68].

#### 3.2.2. Quantitative Evaluation of Antimicrobial Activity

The MIC value is represented by the lowest concentration of the tested BPEs that inhibited microbial growth. The results are presented in Table 7 expressed as µg/mL.

Analysing the quantitative results, it can be noticed that standard strain *S. aureus* ATCC 25422 was the most sensitive Gram-positive bacteria to the influence of BPE samples, with the lowest values of MIC (650 and 380 µg/mL) for P4 and P5 samples.

The lowest MIC values ranged from 270 to 750 µg/mL for Gram-negative bacteria. The most effective antimicrobial extracts were obtained from raw pollen derived from *C. monogyna*, *T. officinale*, *Salix* sp., *Malus* sp., and *Prunus* sp. (P1, P2, and P3). Still, the P3 had the lowest MIC values. It inhibited microbial growth of all Gram-negative bacteria and yeast strains (except on *C. albicans* ATCC 1688 and *C. guillermondii*). P4 and P5 determined sensitivity to *C. albicans* ATCC 1688, *C. guillermondii*, and *C. krusei*. According to previous studies [58,69,70,71,72], antimicrobial activity correlates with these samples’ antioxidant activity.

In other studies, the MIC values of BPEs range from 2 to 2580 µg/mL for *S. aureus*, comparable to market ATBs [18,22,73,74,75,76], while for *P. aeruginosa* they range between 2.47 and 3710 µg/mL [18,22,74,76], as well as between 30,000 and 150,000 µg/g depending on *Pseudomonas* type and the storage conditions of pollen samples [66]. 

#### 3.2.3. Semiquantitative Assessment of Microbial Adherence to the Inert Substratum

The influence of the BPE on the pathogenic microbial strains’ adherence to the inert substratum is presented in Table 8.

According to the MIC and MCBE values (Table 7 and Table 8), *S. aureus* ATCC 25422, *P. aeruginosa* ATCC 25853, and *C. glabrata* were the most sensitive tested strains. The BPEs had a moderate influence on the yeast’s growth. 

P1, P2, and P3 had the lowest values of MCBE on Gram-positive and Gram-negative bacteria. Moreover, these samples presented an inhibitory effect against *C. famata*, *C. glabrata*, *C. krusei*, and *C. lusitaniae*. In the case of *C. albicans*, the P4 and P5 were the most active BPEs; and for *C. guillermondii*, P1, P4, and P5 had moderate antimicrobial activity. 

The most sensitive yeast was *C. glabrata* (the MIC and MCBE values ranged from 540 to 1250 µg/mL), followed by *C. guillermondii* and *C krusei*. A different study confirmed the results for *C. krusei* and *C. glabrata* strains [69,77]. The *C. famata* strain was the most resistant yeast. Still, there are no references to compare our results; these strains’ sensitivity to bee pollen is not tested.

The qualitative antimicrobial assays (Table 6) were confirmed by the MIC and MCBE assays (Table 7 and Table 8) and correlate with the results of the chemical composition analysis (Table 3, Table 4 and Table 5).

According to Singh et al. [78], another hydroalcoholic extract rich in rutin was efficient against some pathogens such as *P. aeruginosa,* which is consistent with our results where P3 has the highest rutin content. Similar results are available in the literature against other pathogens such as *S. aureus* and *E. coli* [79]. Positive results were also obtained for rutin against eight standard strains and their drug-resistant isolates. Some of them were also tested in this paper (*P. aeruginosa*, *S. aureus* and *C. krusei*) [80].

#### 3.2.4. Evaluation of the Inhibitory Effect of the BPE Samples on the Ability of the Tested Microbial Strains to Attach to the Cellular Substrate 

The antimicrobial effect of bioactive compounds from BPEs on the adherence capacity of microbial strains is presented in the figures below. The sterile culture media represented the negative control, and the microbial growth control was represented by (C+).

**Figure 1:**
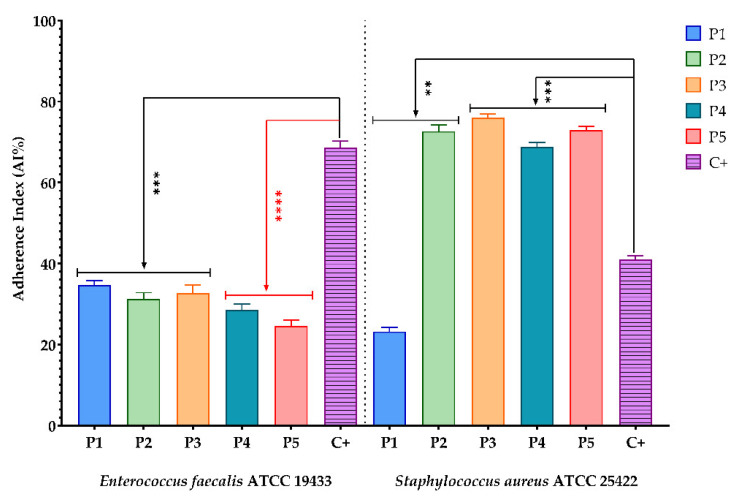
Graphic representation of AI% values representing the influence of BPE on adherence capacity of Gram-positive bacteria; (P1–P5)-BPE samples; (C+)-bacterial adherence control. The differences between antibacterial activity of BPE samples and bacterial adherence control were statistically analysed by performing one-way ANOVA, followed by Dunnett’s multiple comparisons. (** *p* < 0.005; *** *p* < 0.001; **** *p* < 0.0001).

**Figure 2:**
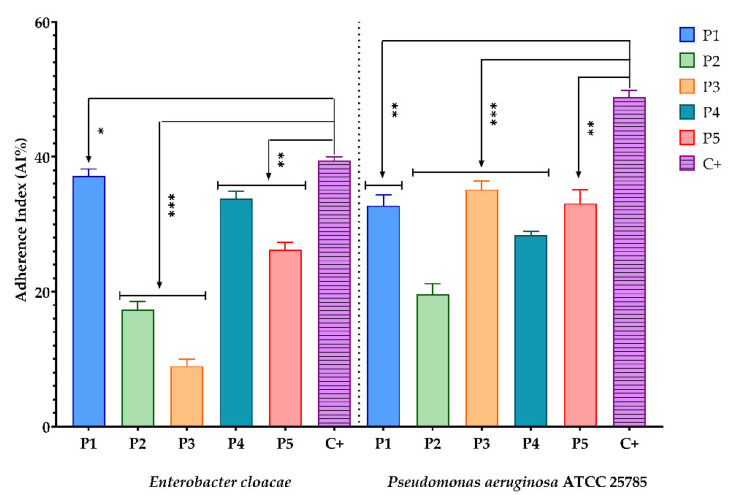
Graphic representation of AI% values representing the influence of BPE on the adherence capacity of Gram-negative bacteria; (P1–P5)-BPE samples; (C+)-bacterial adherence control. The significant impact of the BPE on the adherence capacity of *E. cloacae* and *P. aeruginosa* was tested by performing one-way ANOVA and Dunnett’s multiple comparisons tests. All data were considered statistically significant. (* *p* < 0.05; ** *p* < 0.01; *** *p* < 0.001).

**Figure 3:**
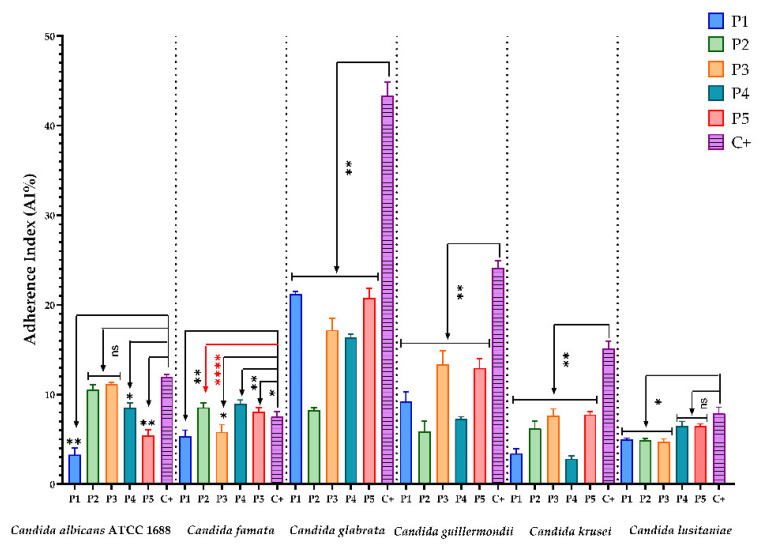
Graphic representation of AI% values representing the influence of BPE on the adherence capacity of yeast strains; (P1–P5)-BPE samples; (C+)-yeast adherence control. The antimicrobial effect of BPE on yeast strains was compared using one-way ANOVA and Dunnett’s multiple comparisons tests. The results were considered statistically significant. (ns—not significant; * *p* < 0.05; ** *p* < 0.01; **** *p* < 0.0001).

Experimental data show that for Gram-negative bacterial strains (*E. cloacae* and *P. aeruginosa* ATCC 25785) and yeasts (*C. albicans* ATCC 1688, *C. glabrata*, *C. guillermondii*, *C. krusei*, and *C. lusitaniae*), all BPEs reduced the adherence capacity compared to the control (Figure 1, Figure 2 and Figure 3). One exception was *C. famata,* with only P1 and P3 samples expressing this effect. Regarding Gram-positive bacterial strains, the same inhibitory effect was observed for *E. faecalis* ATCC 19433. Still, for *S. aureus* ATCC 25422, only P1 inhibits the ability to adhere to the inert substratum. A possible explanation could be related to the ferulic acid content of P1, because this phenolic acid is known for biofilm prevention of *S. aureus* [81]. At the same time, catechin showed great antibacterial activity against this strain [82].

The biological activity of bee pollen depends on the chemical composition profile, mostly flavonoids, phenolic acids, FA, phytosterols and enzymes [83], or *Lactobacillus* strains from raw BP [84,85,86]. The antimicrobial activity of BP is explained by the presence of flavonoids and phenolic compounds in its composition. These compounds mediate in the interruption of the metabolism of bacteria by producing complexes that adhere to and disrupt the cell wall, blocking the ion channels and the electron transport chain, respectively, which determines adenosine triphosphate synthesis [69]. Bridi et al. [21] claim that the evaluation of antimicrobial activity depends on the method applied; for example, in the case of MIC analysis, bioactive compounds in extracts come into contact more easily with microbial strains than disc diffusimetric assays but are not as reproducible. 

Antimicrobial activity of benzoic acids derivates (2,4-dihydroxybenzoic acid, gallic acid, vanillic acid, syringic acid), cinnamic acid derivates (cinnamic acid, *o*/*p*-coumaric acid, caffeic acid, ferulic acid, chlorogenic acid), and some flavonoids (quercetin, rutin, chrysin) was reported in a previous study [87]. Still, the dihydroxybenzoic acids had higher antibacterial activity against Gram-negative and positive bacteria, and the others did not present the inhibitory effect. Borges et al. [88] demonstrated that gallic and ferulic acid had antimicrobial activity against *S. aureus*, *L. monocytogenes*, *E. coli*, and *P. aeruginosa* using MIC and minimum bactericidal concentration evaluation. Additionally, these two acids act irreversibly and destructively on the bacterial membranes by changes in surface hydrophobicity [88]. Sorrentino et al. [89] also reported the antibacterial activity of gallic acid against *Pseudomonas* sp. Furthermore, *S. aureus*, *S. pneumoniae*, *B. subtilis*, *S. typhimurium*, *S. dysenteriae*, and *E. coli* were sensitive to *p*-coumaric acid by leakage of bacterial cell membranes and binding DNA [90]. Some researchers showed a synergic antibacterial effect between caffeic acid and several ATBs against *S. aureus* clinical resistance strains, demonstrating the potential of phenolic compound in treating infections with multidrug resistance strains [91]. 

The flavonoids could penetrate the cell wall membrane due to their amphiphilic properties and induce the antimicrobial effect [92,93]. Adamczak et al. [94] considered that Gram-negative bacteria were more sensitive than Gram-positive bacteria to flavonoids. Still, apigenin, chrysin, and luteolin have no antibacterial activity against these strains [94]. The most sensitive strain was *E. coli*, followed by *P. aeruginosa*, *E. faecalis,* and *S. aureus*. The *P. aeruginosa* was resistant to kaempferol, quercetin, chlorogenic acid, and apigenin but moderately affected all strains. The antifungal potential of kaempferol and quercetin was reported by Gadelha Rocha et al. [95] after MIC and biofilm assays of these flavonoids against some *Candida* sp. Other papers show the antimicrobial activity of isorhamnetin, pinocembrin, quercetin, and kaempferol on *S. aureus*, *E. coli*, *Salmonella* sp., and *C. albicans* [96,97,98]. Additionally, galangin could be used to treat multidrug-resistance bacteria, such as vancomycin-intermediate *S. aureus*, because it presents an inhibitory effect of murein hydrolase activity and the development of Gram-positive bacteria [99]. 

Furthermore, the antimicrobial activity of BPE could be attributed to some compounds identified by GC-MS. Thus, from the compounds present in all BPE samples tested, 7,9-di-tert-butyl-1-oxaspiro (4,5) deca-6,9-diene-2,8-dione is a flavonoid that has previously shown significant antimicrobial properties [100,101]. At the same time, *β*-ionone was responsible for inhibitory activity on some strains of *E. coli* and *C. albicans* [100,101]. 

The antibacterial properties of some volatile oils isolated from various plants (*Helichrysum rugulosum*, *Eugenia umbelliflora*, *Salvia officinalis*) against *S. aureus* have been attributed mainly to globulol, present only in P3 and P5 [102].

In addition, methoxy-eugenol, present only in P5, has a known inhibitory effect on efflux pumps (due to methoxy grouping) and may cause a more significant intracellular accumulation of ATBs in the bacterial cell [103]. 

The BPE’s antimicrobial activity is deeply linked to phenolic content [104]. Still, other compounds seem to have their contribution, such as fatty acids, exosome-like vesicles, and possible microbial metabolites [16]. Casillas-Vargas et al. [105] consider that FA is the new generation of antibacterial agents helpful in treating infections caused by Gram-positive and Gram-negative bacteria, given the studies on their mechanisms of action in recent years. Thus, the literature mentions both classical mechanisms of action for them (such as those of ATBs: inhibition of cell wall, inhibition of protein synthesis, disruption of the cytoplasmic membrane, inhibition of DNA/RNA replication, and inhibition of some metabolic pathways) and unconventional ones that may have the advantage of decreasing bacterial resistance to ATB or reducing virulence (such as inhibition of horizontal gene transfer, quorum sensing, and efflux pumps) [105].

#### 3.2.5. Evaluation of the Prebiotic Effect of the BPE Samples on the Ability of Two Microbial Strains with Probiotic Potential to Adhere to a Cellular Substrate

The relationship of prebiotics with gut microbiota plays an essential role in improving human health. They can modulate the gut microbiota’s composition, growth, and population [106]. The prebiotic effect of the BPE was examined using Hep-2 cell lines. Good probiotic functionality is considered if the probiotic strains adhere to mucus or intestinal epithelial cells. The interaction between epithelial cells and probiotic bacteria requires complex cross-talk mechanisms involving physical interactions between bacteria and Hep-2 epithelial cells, adhesions, complementary receptors, and the modulation of pro-inflammatory and anti-inflammatory molecules [50]. 

**Figure 4:**
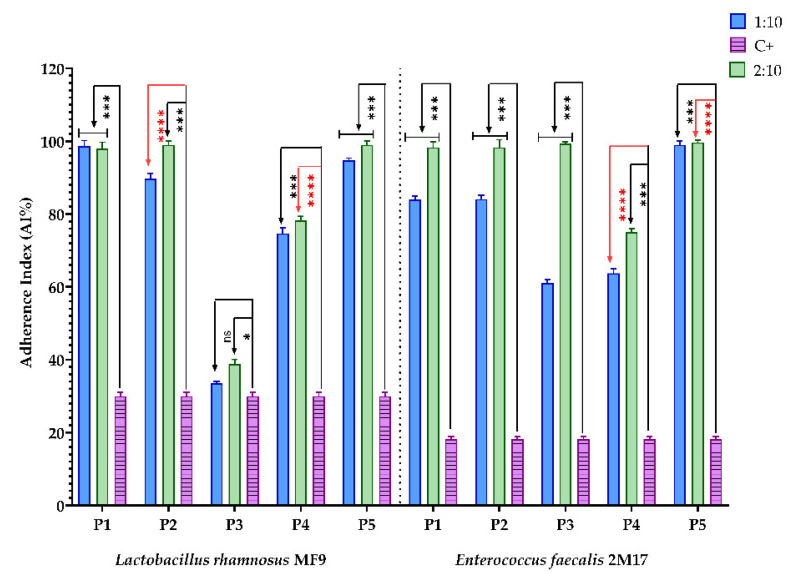
Graphic representation of AI% values representing the influence of BPE on the adherence capacity of the bacterial strains with probiotic potential; (P1–P5)-BPE samples; (C+)-bacterial adherence control. The significant impact of the effect of BPE on lactic strains was statistically analysed through one-way ANOVA and Dunnett’s multiple comparisons tests. (ns—not significant; * *p* ≤ 0.01; *** *p* < 0.001; **** *p* < 0.0001).

All BPEs stimulated the adherence capacity (Figure 4) of the microbial strains with probiotic potential, *L. rhamnosus* MF9 and *E. faecalis* 2M17, compared to the control, at 1:10 and 2:10. The adherence capacity of *E. faecalis* 2M17 was enhanced in the presence of P3. Additionally, the P3 highly determined sensitivity against pathogenic strains, according to MIC and MCBE results (Table 7 and Table 8). Thus, it can be concluded that P3 can ensure adherence to *E. faecalis* 2M17 and inhibit microbial strains, which can be correlated with rebiosis. 

Moreover, all BPE tested samples have a benefic action because they significantly improved the adherence capacity of both probiotic strains, except P3. In this case, P3 significantly influenced the adherence capacity of *E. faecalis* 2M17. Still, against *L. rhamnosus* MF9, the AI% is not significant or minor, depending on the dilution. 

In previous papers, Leite de Souza et al. [107] presented that *L. acidophilus* ATCC 1643 showed a higher capacity to adhere to intestinal cells in the presence of purified phenolic compounds: rutin, epicatechin, chlorogenic acid, quercetin, and *p*-coumaric acid. Parkar et al. [108] showed that rutin increased *L. rhamnosus* adherence to Caco-2 epithelial intestinal cells. In another study, Boubakeur et al. [109] determined the positive effect on growth of *L. rhamnosus* and *S. thermophilus* under the influence of several flavonoids. Furthermore, they proved antibacterial activity separately for these flavonoids against *S. aureus* and *E. coli*.

#### 3.2.6. Assessment of the Prebiotic Effect of the BPE on the Growth for Two Bacterial Strains with Probiotic Potential 

**Figure 5:**
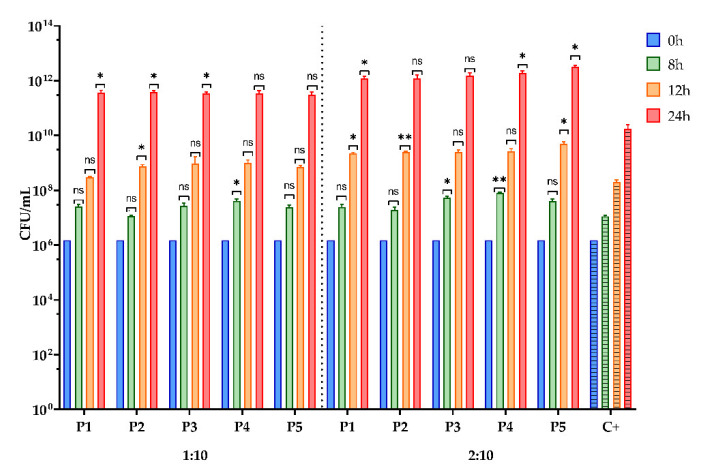
Graphic representation of CFU/mL values representing the influence of BPE on the bacterial growth of *Lactobacillus rhamnosus* MF9; (P1–P5)-BPE samples; (C+)-*L. rhamnosus* MF9 growth *control*. The prebiotic effect of BPE on *L. rhamnosus* MF9 growth was statistically analysed in one-way ANOVA and Dunnett’s multiple comparisons tests. The data is considered statistically significant. (ns—not significant; * *p* < 0.05; ** *p* < 0.007).

**Figure 6:**
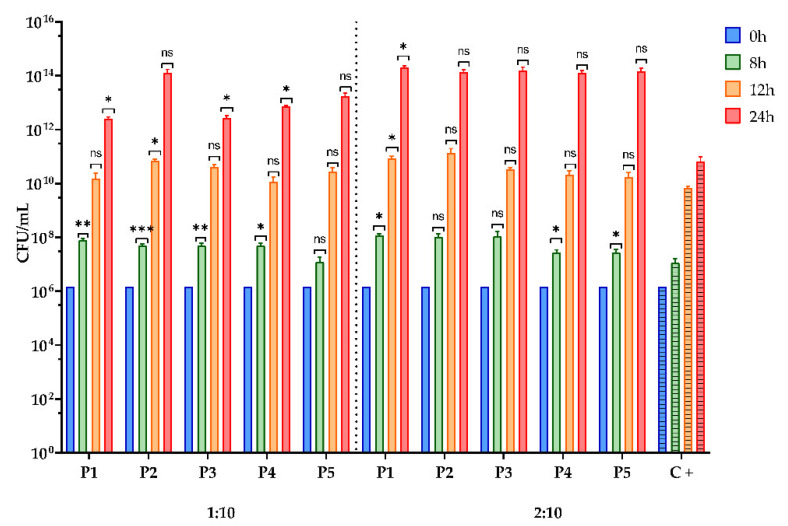
Graphic representation of CFU/mL values representing the influence of BPE on the bacterial growth of *Enterococcus faecalis* 2M17; (P1–P5)-BPE samples; (C+)-*E. faecalis* 2M17 growth control. The prebiotic effect of BPE on *E. faecalis* 2M17 growth was statistically analysed in one-way ANOVA and Dunnett’s multiple comparisons tests. The data is considered statistically significant. (ns—not significant; * *p* < 0.05; ** *p* < 0.08; *** *p* = 0.0009).

The prebiotic compounds are considered selective substrates that stimulate probiotic bacteria, and some of them, such as polyphenols, can improve the production of FA [110]. Bee pollen can be considered an essential source of prebiotic compounds, and the effect of the BPE samples on the growth of *L. rhamnosus* MF9 and *E. faecalis* 2M17 is presented in Figure 5 and Figure 6.

Regarding the prebiotic effect of the BPE on the growth curve for tested probiotic strains, all BPEs stimulated the growth of the microbial strains with probiotic potential compared to the control, at both dilution 1:10 and 2:10 (Figure 5 and Figure 6). It is worth mentioning that the beneficial activity can be visualised even at 8 and 12 h, but especially at 24 h when 2–3 orders higher CFU/mL values are obtained in the presence of the BPE compared to the control. Sánchez-Maldonado et al. [111] demonstrated that lactobacilli adjust to phenolic compounds and can metabolise them, with some exceptions. The lipophilicity of hydroxybenzoic acids influences antimicrobial activity more than hydroxycinnamic acids [111]. Likewise, Alberto et al. [112] showed that gallic acid and catechin could be considered growth-promoting agents of lactobacilli (*L. hilgardii* 5 w). P5 has the highest concentration of gallic acid (2.310 µg/g BP), while P2 has the second highest value of catechin concentration (0.587 µg/g BP). A recent study has shown that *L. rhamnosus* GG ATCC 53103 and *L. acidophilus* NRRLB 4495 better adapt to catechin and gallic, protocatechuic, and vanillic acids than the pathogenic bacteria tested [28]. 

According to Guldas [113], the probiotic bacterial counts increased with the concentration of bee pollen up to 75 mg during 24 h of cultivation (*B. animalis* with 8.09%, *L. acidophilus* with 8.70%, *L. casei* with 10%) and generally above this concentration remained unchanged. Adding bee pollen stimulated lactic acid production and bacterial growth, especially on *L. acidophilus*. The bee pollen also contains significant food sources for probiotic bacterial growth, such as glucose and fructose, B vitamins and derivates, and trace elements such as Fe, Cu, Zn, and Mn [113]. 

#### 3.2.7. Assessment of the Synergic Influence of BPE and Probiotic Soluble Compounds on the Capacity of Some Pathogenic Strains to Adhere to the Cellular Substratum

**Figure 7:**
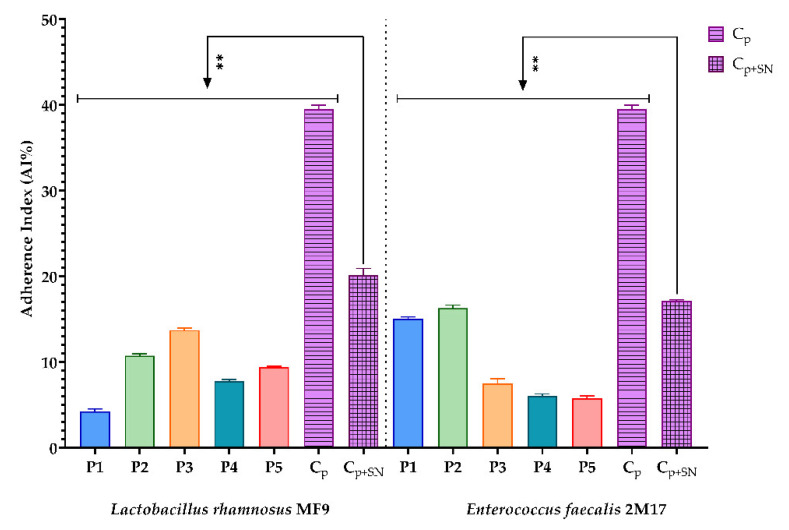
Graphic representation of AI% values representing the synergic influence of BPE and probiotic Soluble Compounds (SN) on the adherence capacity of *Enterobacter cloacae*; (P1–P5)-BPE samples, pathogenic strains adherence control (Cp), pathogenic strains with probiotic supernatant adherence control (C_P+SN_). The differences between the effects of BPE and soluble compounds of strains on adherence capacity of clinical strains were statistically analysed using one-way ANOVA, followed by Dunnett’s test for multiple comparisons of groups. (** *p* < 0.01).

**Figure 8:**
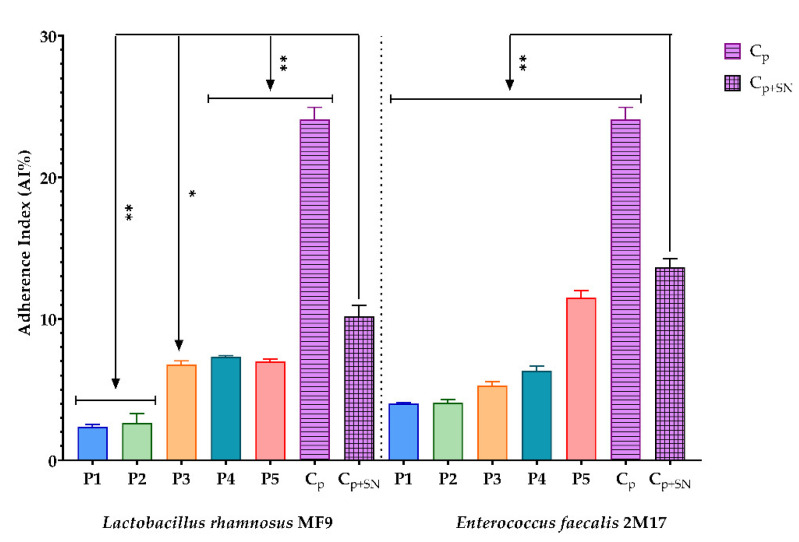
Graphic representation of AI% values representing the synergic influence of BPE and probiotic Soluble Compounds (SN) on the adherence capacity of *Candida guillermondii;* (P1–P5)-BPE samples, pathogenic strains adherence control (Cp), pathogenic strains with probiotic supernatant adherence control (C_P+SN_). The differences between the effects of BPE and soluble compounds of strains on the adherence capacity of clinical yeast strains were statistically analysed using one-way ANOVA. Furthermore, Dunnett’s test for multiple comparisons of groups was performed. The results were considered statistically significant. (* *p* < 0.05; ** *p* < 0.009).

Besides the assessment of the antimicrobial effect of the BPE and soluble compounds of lactic strains on the adherence capacity to the sensitive cellular substrate of some pathogenic strains, it can be considered that the BPE, together with the soluble compounds of *L. rhamnosus* MF9 and *E. faecalis* 2M17, inhibited without exception the adherence capacity of the *C. guillermondii* and *E. cloacae*, compared to the control at 1:10 (Figure 7 and Figure 8). 

According to the results obtained in this study, the BPEs, rich in compounds with prebiotic effects, strongly stimulated the growth of probiotic strains. Furthermore, when they were in contact with the soluble compounds of the two lactic strains, they determined a synergistic inhibitory effect on the multiplication process of the two clinical strains with pathogenic potential. To our knowledge, there are no data available in the literature to confirm this. 

In the literature conflicting results are presented regarding the influence of phenols on lactobacilli. For example, some researchers consider that unabsorbed polyphenols and their metabolites can stimulate *Lactobacillus* sp. growth [114,115,116]. In contrast, others show that some polyphenols have concentration-dependent inhibitory effects on *L. plantarum* growth and reduce the capacity of probiotic *L. rhamnosus* to adhere to intestinal cell lines [117,118]. Some species or strains of *Lactobacillus* sp. may be more susceptible to polyphenols than others. Cheng et al. [119] showed that a *Schisandra chinensis* BPE decreased the relative abundance of pathogenic bacteria and stimulated the *Lactobacillus* strain from gut microbiota in obese mice. The protective effect of BP on gastrointestinal infections in broiler chickens [120] and diseases related to induced burns in swine was also studied [121]. In the first case, BP was administered via food supplements, and in the second case, extracts or pharmaceutical preparations based on BP were used. As a result, there was a beneficial strain reduction of *Enterobacteriaceae* in the gastrointestinal tract for broiler chickens and the prevention of infection in pigs, respectively. At the same time, in both studies, the animals had a better evolution, with a better state of health than the control groups [122]. 

### 3.3. Cytototoxic Activity of BPEs 

**Figure 9:**
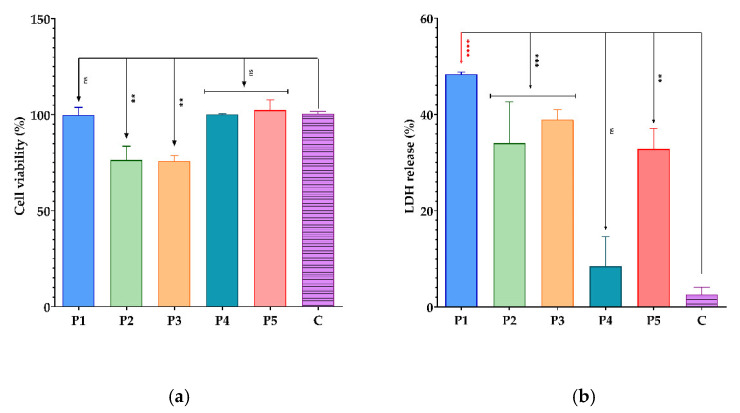
(**a**) Graphic representation of the absorbance values to evaluate the influence of BPEs on cell viability, the proliferation of tumoural cells (MTT assay). (**b**) Graphic representation of the absorbance values to evaluate the cytotoxic effect of BPEs on tumoural cells (LDH assay); (P1–P5) BPE samples, C-cell culture control. The differences between cell viability and proliferation for the MTT assay were statistically analysed using one-way ANOVA followed by Dunnett’s multiple comparisons tests. (ns—not significant; ** *p* < 0.007); The LDH data were statistically analysed in the same way. (ns—not significant; ** *p* = 0.0013; *** *p* ≤ 0.0009; **** *p* < 0.0001).

The in vitro tumour cell viability and proliferation in the presence of BPEs were examined using a colourimetric assay by quantitatively evaluating living cells in culture. The principle of the MTT method is based on the conversion of MTT (yellow) by mitochondrial dehydrogenase enzyme (from living cells) to formazan (purple) [123,124]. The viability is expressed as the percentage of living cells compared to control (Figure 9a). 

The cytotoxic potential of all BPEs on tumour cells was evaluated by LDH assay. Damage to the cell membrane causes the leak of LDH (cytosolic enzyme from the cytoplasm) into the extracellular fluid [125]. In vitro release of LDH indicates the number of dead cells in the culture, expressed as a percentage compared to control (Figure 9b).

The MTT analysis showed that all BPEs did not stimulate the proliferation of Hep-2 cells (Figure 9a). P3 has the lowest percentage of cell viability (75.90%), showing an inhibitory effect, followed by P2 (76.34%). These results are confirmed by the LDH level that was increased in the presence of these two samples (Figure 9b).

The P5 sample stimulated the proliferation of Hep-2 cells (102.6%), but the stimulatory effect was not significant. Among the tested BPEs, P4 had the lowest level of cytotoxicity, as shown by the quantification of LDH. Conversely, P1 has the highest LDH levels (Figure 9a) but did not significantly influence the viability and the proliferation of tumoural cells, these results being contradictory. Otherwise, P2 and P3 presented the best cytotoxic effects on tested tumour cells and correlated with the previous results; they can be considered potential tumour proliferation inhibitory agents. Abu Shady et al. [126] demonstrated the antitumour activity of BPEs was higher against human hepatocellular carcinoma cells (HepG2) than human breast adenocarcinoma cells (MCF-7). Furthermore, Uçar et al. [127] presented in their study that bee pollen and propolis extracts increase apoptosis of MNC and HL-60 myeloid cancer cells. Arung et al. [128] showed the antitumour activity of honey, bee pollen and propolis extracts against MCF-7, Caco-2, and Hela (*human cervical adenocarcinoma*) cell lines. 

The cytotoxic effects of BPEs on tumour cells can be explained by the presence of bioactive compounds in BP. For example, P3 presented the highest levels of rutin and the pronounced inhibitory effect on tumoural cells. This flavonoid acts as a modulator of molecular mechanisms that trigger tumour cells (apoptosis, inflammation, angiogenesis, autophagy, etc.). Additionally, the antitumour potential of rutin was demonstrated against various cancer types (colon, hepatocellular, gastric, breast, adenocarcinoma, cervical, etc.) [129,130,131,132]. Kalinowska et al. [133] reported the anticancer activity of several hydroxybenzoic acids in two human breast cancer cell lines (MCF-7 and MDA-MB-231).

Furthermore, the inhibitory effect of free FA (saturated and unsaturated) against different tumour cell lines is presented by Jóźwiak et al. [134] and considered that saturated FA with carbon chains >C_10_ does not present antitumour activity. For unsaturated FA, the inhibitory effect increases with the level of unsaturation. Furthermore, a higher inhibitory effect against different tumour cell lines when FAs were conjugated with chemotherapeutics drugs was reported. 

## 4. Conclusions

Bee pollen is an excellent source of phenolic compounds responsible for antioxidant activity and fatty acids, including polyunsaturated and terpenic compounds. The main purpose of this study was to evaluate the relationship between the chemical composition, antioxidant properties, effect on the growth of selected probiotic and pathogenic bacteria, and the cytotoxic effects of BPEs on the tumour cell line. 

P1, P2, and P4 samples had higher phenol and flavonoid content and the best antioxidant activity. Each BPE sample had its own phenolic profile. Several phenols were present in significant quantities: 4-hydroxybenzoic acid (P2), chlorogenic acid (P4), ferulic acid (P1, P2), and gallic acid (P5). Regarding flavonoids, rutin (P3 > P4 > P1), quercetin (P4 > P5), kaempferol (P5 > P4 > P3), and isorhamnetin (P5 > P3) were prevalent. Some terpenes were identified as minor constituents (globulol, methyleugenol, etc.). FA was in higher percentages, such as stearic acid methyl ester (P4), linolenic acid (P3, P5), and linoleic acid (P1). 

All BPEs presented antimicrobial activity against pathogenic strains. The Gram-positive bacteria were more sensitive than Gram-negative bacteria and yeasts. Antibacterial activity seems to be given to the chemical composition of BP, and a synergistic effect may be responsible in some cases for antimicrobial activity. Therefore, it is difficult to accurately attribute antimicrobial activity to a single compound/class of compounds because we refer to samples with complex, multi-component matrices that encompass compounds with different polarities, peculiar percentages, and variable provenance.

Phenolic compounds from the studied extracts could be a rich source of prebiotic compounds, given the stimulating effect of the growth of microbial strains with probiotic potential (*L. rhamnosus* MF9 and *E. faecalis* 2M17). Furthermore, a synergistic antimicrobial effect of the BPEs was observed along with soluble compounds of *L. rhamnosus* MF9 and *E. faecalis* 2M17 against two clinical pathogenic strains. Additionally, all BPEs did not stimulate the proliferation of Hep-2 cells, and P2 and P3 samples presented a higher inhibitory effect. Furthermore, the antitumour activity of P2 and P3 is confirmed by cytotoxicity analysis. 

These results indicate the potential of bee pollen to be used as an antimicrobial, prebiotic, and tumour proliferation inhibitory agent that, in association with probiotic compounds, maintains and even improves gut homeostasis by promoting the recovery of intestinal microbiota (rebiosis), fighting or preventing bacterial infections, and inhibiting the onset of tumour processes. Further work must be done to assess whether these extracts can be loaded in specific supports (including pH-sensitive polymers, porous silica-based materials, etc.) and assure a targeted delivery at the colon level, protecting these agents from the harsh conditions of the stomach. 

## Figures and Tables

**Table 1:** Origin of bee pollen samples.

Sample	Flowering Plants	Date of Harvest
P1	*Cornus mas*, *Corylus avellana*, *Armeniaca vulgaris*, *Prunus cerasifera*, *Salix* sp., *Colchicum autumnale*, *Taraxacum officinale*, *Viola odorata*, *Helleborus* sp. etc.	1 April 2020
P2	*Cornus mas*, *Corylus avellana*, *Armeniaca vulgaris*, *Prunus cerasifera*, *Ribes* sp., *Salix* sp., *Taraxacum officinale*, *Viola odorata* etc.	10 April 2020
P3	*Prunus domestica*, *Malus* sp., *Cerasum* sp. etc.	24 April 2020
P4	*Malus* sp., *Pyrus* sp., *Cydonia oblonga*, *Crataegus monogyna*, *Fraxinus excelsior* etc.	1 May 2020
P5	*Robinia pseudocacia*, *Rosa centifolia*, *Rosa canina*, *Cornus sanguinea*, *Acer campestre* etc.	1 June 2020

**Table 2:** The moisture content.

Sample	Moisture (%) ± SD
P1	27.83 ± 0.01 ^a^
P2	22.62 ± 0.02 ^a^
P3	26.47 ± 0.01 ^a^
P4	31.91 ± 0.02 ^a^
P5	35.87 ± 0.02 ^a^

We compared the differences between samples using RM one-way ANOVA and Tukey’s multiple comparisons test. The *p*-value for all samples was <0.0001 and is represented in the table by ^a^.

**Table 3:** Total phenol content, total flavonoid content, and antioxidant activity.

Sample	TPC (GAE) ^1^	TFC (QE) ^2^	TEAC ^3^
**P1**	**15.51 ± 0.01 ^c,d^**	**0.27 ± 0.01 ^d^**	**0.06 ± 0.02 ^b^**
**P2**	**16.15 ± 0.02 ^c,d^**	**0.30 ± 0.01 ^d^**	**0.07 ± 0.01 ^b,d^**
P3	13.24 ± 0.01 ^d^	0.20 ± 0.02 ^d^	0.04 ± 0.02 ^d^
**P4**	**14.46 ± 0.01 ^d^**	**0.26 ± 0.01 ^d^**	**0.05 ± 0.01 ^b^**
P5	10.77 ± 0.02 ^d^	0.19 ± 0.01 ^d^	0.03 ± 0.01 ^a,d^

^1^ TPC expressed as mg gallic acid/g BP; ^2^ TFC expressed as mg quercetin/g BP; and ^3^ TEAC expressed as mmol Trolox/g BP. We compared differences between the resulting TPC, TFC, and antioxidant assays of all BPEs using one-way ANOVA and Tukey’s multiple comparisons tests. ^a, b, c^ and ^d^ letters indicate significant differences between samples (^a^ *p* < 0.05; ^b^ *p* < 0.007; ^c^ *p* = 0.0001; ^d^ *p* < 0.0001).

**Table 4:** The phenolic acid and flavonoid concentrations from BP (µg/g).

Phenolic Compound	Sample				
	P1	P2	P3	P4	P5
**Phenolic Acids**					
gallic acid	0.015	0.087	0.157	0.075	**2.310**
3,4-dihydroxybenzoic acid	0.254	0.401	0.454	0.209	**0.549**
4-hydroxybenzoic acid	7.603	**19.770**	8.455	4.458	2.685
chlorogenic acid	0.733	2.441	6.481	**46.939**	0.275
caffeic acid	0.404	0.471	0.734	**1.167**	0.275
syringic acid	0.090	ND	ND	ND	**0.325**
*p*-coumaric acid	1.227	1.151	1.153	**1.256**	1.036
ferulic acid	**2.978**	**2.894**	0.961	1.271	1.199
cinnamic acid ^#^	0.898	0.227	**2.236**	1.286	0.400
**Flavonoids**					
epicatechin	0.868	1.029	0.070	**1.286**	0.137
catechin	**1.257**	0.959	0.332	0.224	0.587
rutin	45.662	1.691	**135.301**	69.451	ND
myricetin	**0.943**	0.506	0.052	0.224	0.012
quercetin	3.577	1.639	3.214	**7.883**	**5.981**
kaempferol	2.155	1.308	7.337	8.676	**26.472**
isorhamnetin	3.502	0.558	12.141	5.086	**40.220**
apigenin	0.015	0.052	0.070	0.165	**2.735**
pinocembrin	0.644	**2.197**	0.559	1.227	0.412
galangin	ND	**0.506**	0.017	0.239	0.175
chrysin	0.464	**1.970**	0.384	0.853	0.387

ND (not detected) Naringenin, hesperidin, pinostrombin, and resveratrol were not detected in the bee pollen samples. ^#^ *p*-coumaric, ferulic, caffeic, and chlorogenic acids are derivatives of cinnamic acid (which does not contain phenolic groups), and for this reason it was still included in the table.

**Table 5:** Processed BPE composition.

Compounds	RI ^a^	RA ^b^ (%)				
		P1	P2	P3	P4	P5
Isophorone	1132	ND	ND	ND	ND	**0.32**
Lilac aldehyde A	1149	**0.12**	**0.17**	ND	ND	ND
Lilac aldehyde B	1159	**0.27**	**0.20**	ND	ND	ND
Lilac aldehyde D	1174	**0.11**	**0.16**	ND	ND	ND
Caprylic acid	1181	ND	ND	**0.17**	ND	ND
Lilac alcohol B	1222	**0.15**	**0.45**	ND	ND	ND
Lilac alcohol D	1237	**0.09**	**0.19**	ND	ND	ND
5-Hydroxymethylfurfural	1245	ND	ND	**0.44**	ND	ND
*β*-Ionone	1499	**0.25**	**0.42**	**0.15**	**0.07**	**0.53**
2,4-Di-tert-butylphenol	1521	**0.36**	**0.45**	**0.57**	**0.12**	**0.75**
Dodecanoic acid	1568	ND	ND	**0.23**	ND	ND
Globulol	1572	ND	ND	**0.19**	ND	**0.36**
Methoxyeugenol	1619	ND	ND	ND	ND	**0.42**
Myristic acid	1762	ND	**0.48**	ND	**0.14**	**0.83**
Benzoic acid, phenylmethyl ester	1787	ND	ND	**0.24**	**0.11**	**0.00**
Myristic acid, isopropyl ester	1824	**0.19**	**0.38**	**0.22**	ND	**0.71**
Pentadecanoic acid ethyl ester	1881	**0.17**	**0.14**	**0.66**	ND	ND
7,9-Di-tert-butyl-1-oxaspiro(4,5)deca-6,9-diene-2,8-dione	1940	**0.88**	**1.34**	**0.64**	**0.42**	**2.34**
Palmitic acid	1968	**2.92**	**13.53**	**3.65**	**0.79**	**5.82**
Palmitic acid, ethyl ester	1996	**4.46**	**2.66**	**1.08**	**0.25**	**0.86**
9,12,15-Octadecatrienoic acid, methyl ester	2084	ND	ND	**2.28**	ND	ND
Linoleic acid, methyl ester	2090	ND	ND	**2.98**	ND	ND
Linolenic acid, methyl ester	2096	**12.92**	**19.24**	**25.87**	**2.10**	**9.02**
Linolenic acid	2107	**14.52**	**24.03**	**43.23**	**6.08**	**43.42**
Linoleic acid	2112	**19.40**	**4.74**	**4.92**	**0.59**	**3.40**
Linolenic acid, methyl ester	2119	**14.27**	**3.82**	**9.84**	**1.01**	**6.12**
Stearic acid, methyl ester	2126	**27.51**	**12.58**	ND	**88.31**	**10.64**
Stearic acid, ethyl ester	2222	ND	**15.04**	ND	ND	**5.10**
Unidentified compounds	-	1.41	0.00	2.36	0.00	9.34
**Total**	-	**98.59**	**100.00**	**97.37**	**100.00**	**90.66**
Fatty acids and esters	-	**96.36**	**96.64**	**95.13**	**99.28**	**85.93**
Terpenes and terpenoides	-	**0.25**	**0.42**	**0.34**	**0.07**	**1.32**

ND (not detected) ^a^ RI = Kovats index, measured relative to n-alkanes (C_8_–C_20_) on a DB-5MS capillary column; ^b^ RA = relative area (%) = relative contents expressed as percentages of the total compounds.

**Table 6:** The growth inhibition zone diameters (GIZD).

Strain	GIZD (mm)					
	P1	P2	P3	P4	P5	C_Et_
**Gram-Positive Bacteria**						
1. *Enterococcus faecalis* ATCC 19433	**14.00** **± 1.00 ^d^**	13.50 ± 0.50 ^b^	11.50 ± 0.50 ^a^	**14.40** **± 0.20 ^d^**	11.50 ± 0.50 ^b^	7.00 ± 1.00
2. *Staphylococcus aureus* ATCC 25422	13.00 ± 0.50 ^b^	13.00 ± 1.00 ^a^	12.00 ± 0.23	13.00 ± 0.35 ^b^	**14.00** **± 0.52 ^c^**	9.70 ± 0.60
**Gram-Negative Bacteria**						
1. *Enterobacter cloacae*	**15.00** **± 0.80 ^b^**	14.30 ± 0.57 ^a^	13.30 ± 0.25	13.00 ± 0.06	14.00 ± 0.45 ^a^	12.00 ± 1.00
2. *Escherichia coli* ATCC 25923	14.50 ± 0.50 ^a^	**16.00 ± 0.50** ^b^	14.90 ± 0.90 ^a^	14.40 ± 0.20 ^a^	14.50 ± 0.50 ^a^	12.70 ± 0.60
3. *Pseudomonas aeruginosa* ATCC 25785	**12.00 ± 0.50 ^a^**	**12.70** **± 0.30 ^a^**	10.90 ± 0.15	10.80 ± 0.15	**12.00** **± 0.25 ^a^**	9.70 ± 0.60
**Yeasts**						
1. *Candida albicans* ATTC 1688	15.10 ± 0.15	14.70 ± 0.07	15.10 ± 0.17	**16.00** **± 0.06 ^b^**	**15.70** **± 0.05 ^a^**	14.40 ± 0.15
2. *Candida famata*	**17.60** **± 0.70 ^a^**	**17.00** **± 0.40 ^a^**	**17.40** **± 0.40 ^a^**	16.90 ± 0.15	16.20 ± 0.50	16.50 ± 0.50
3. *Candida glabrata*	**15.50** **± 0.50 ^a^**	**15.00** **± 0.30 ^a^**	14.50 ± 0.25	14.50 ± 0.25	14.40 ± 0.17	14.00 ± 1.00
4. *Candida guillermondii*	**18.10** **± 0.17 ^c^**	17.50 ± 0.05 ^b^	17.10 ± 0.15 ^a^	16.90 ± 0.11 ^b^	**18.50** **± 0.10 ^c^**	16.00 ± 0.05
5. *Candida krusei*	16.20 ± 0.25 ^a^	16.40 ± 0.10 ^a^	16.90 ± 0.15 ^a^	**18.10** **± 0.10 ^b^**	16.00 ± 0.25 ^a^	12.50 ± 0.50
6. *Candida lusitaniae*	**17.80** **± 0.28 ^a^**	16.50 ± 0.10 ^a^	16.50 ± 0.25 ^a^	15.50 ± 0.25	16.10 ± 0.10 ^a^	14.50 ± 0.50

The significant impact of the BPEs on each microbial strain was statistically analysed by one-way ANOVA and Dunnett’s multiple comparisons post hoc test. The resulting data were statistically significant. Different letters indicate the significant differences between samples and C_Et_ (^a^ *p* < 0.05; ^b^ *p* < 0.01; ^c^ *p* < 0.001; ^d^ *p* < 0.0001).

**Table 7:** Determination of MIC values (µg/mL).

Strain	MIC				
	P1	P2	P3	P4	P5
**Gram-Positive Bacteria**					
1. *Enterococcus faecalis* ATCC 19433	**1250**	2150	4290	2510	3000
2. *Staphylococcus aureus* ATCC 25422	1250	1080	2150	**630**	**380**
**Gram-Negative Bacteria**					
1. *Enterobacter cloacae*	2510	2150	**540**	2510	**750**
2. *Escherichia coli* ATCC 25923	1250	**540**	**540**	1250	1500
3. *Pseudomonas aeruginosa* ATCC 25853	**630**	**540**	**270**	1250	1500
**Yeasts**					
1. *Candida albicans* ATTC 1688	5010	2150	4290	**630**	**750**
2. *Candida famata*	**1250**	2150	**1070**	2510	3000
3. *Candida glabrata*	**630**	**540**	1070	1250	3000
4. *Candida guillermondii*	**630**	2150	2150	1250	**750**
5. *Candida krusei*	2510	1080	**270**	1250	3000
6. *Candida lusitaniae*	1250	2150	**270**	2510	3000

**Table 8:** Determination of minimal concentration for biofilm eradication (MCBE) values (µg/mL).

Strain	MCBE				
	P1	P2	P3	P4	P5
**Gram-Positive Bacteria**					
1. *Enterococcus faecalis* ATCC 19433	**1250**	2.150	2150	2510	3000
2. *Staphylococcus aureus* ATCC 25422	1250	1080	2150	**1250**	**750**
**Gram-Negative Bacteria**					
1. *Enterobacter cloacae*	1250	1080	**1070**	2510	1500
2. *Escherichia coli* ATCC 25923	2510	**270**	**540**	1250	1500
3. *Pseudomonas aeruginosa* ATCC 25853	**630**	**540**	270	1250	1500
**Yeasts**					
1. *Candida albicans* ATTC 1688	2510	2150	2150	**630**	**750**
2. *Candida famata*	**1250**	2150	**1070**	2510	3000
3. *Candida glabrata*	**630**	**540**	1070	1250	3000
4. *Candida guillermondii*	**630**	2150	2150	1250	**750**
5. *Candida krusei*	2510	1080	**270**	1250	1500
6. *Candida lusitaniae*	630	2150	**1070**	2510	3000

## Data Availability

Data is contained within the article.

## Supplementary Materials

The following are available online at https://www.mdpi.com/article/10.3390/antiox11050959/s1, Figure S1: TIC and the extracted chromatograms of the main phenolic compounds identified in BPE.
