# Supplementary material for: Bee Pollen Extracts: Chemical Composition, Antioxidant Properties, and Effect on the Growth of Selected Probiotic and Pathogenic Bacteria
Source: Antioxidants (Basel). 2022 May 12;11(5):959. doi: 10.3390/antiox11050959 (PMC9137718; doi:10.3390/antiox11050959)
Supplement: Supplementary file 1 [file antioxidants-11-00959-s001.zip › antioxidants-1696408-supplementary-done.pdf]

*antioxidants*

MDPI

*Article*

# Bee Pollen Extracts: Chemical Composition, Antioxidant Properties, and Effect on the Growth of Selected Probiotic and Pathogenic Bacteria

**Cornelia-Ioana Ilie [1,2], Eliza Oprea [3,4,*], Elisabeta-Irina Geana [5], Angela Spoiala [1,2], Mihaela Buleandra [6], Gratiela Gradisteanu Pircalabioru [7], Irinel Adriana Badea [6], Denisa Ficai [2,8], Ecaterina Andronescu [1,2,9], Anton Ficai [1,2,9,*] and Lia-Mara Ditu [3,7]**

1 Department of Science and Engineering of Oxide Materials and Nanomaterials, Faculty of Chemical Engineering and Biotechnologies, University Politehnica of Bucharest, 1–7 Gh. Polizu Street, 011061 Bucharest, Romania; cornelia_ioana.ilie@upb.ro (C.-I.I.); angela.spoiala@upb.ro (A.S.); ecaterina.andronescu@upb.ro (E.A.)
2 National Centre for Micro and Nanomaterials and National Centre for Food Safety, Faculty of Chemical Engineering and Biotechnologies, University Politehnica of Bucharest, 313 Splaiul Independentei, 060042 Bucharest, Romania; denisa.ficai@upb.ro
3 Department of Microbiology and Immunology, Faculty of Biology, University of Bucharest, 1–3 Aleea Portocalelor, 060101 Bucharest, Romania; lia-mara.ditu@bio.unibuc.ro
4 Department of Organic Chemistry, Biochemistry and Catalysis, Faculty of Chemistry, University of Bucharest, 030018 Bucharest, Romania
5 National R&D Institute for Cryogenics and Isotopic Technologies—ICIT, 4th Uzinei Street, 240050 Râmnicu Vâlcea, Romania; irina.geana@icsi.ro
6 Department of Analytical Chemistry, Faculty of Chemistry, University of Bucharest, 90–92 Șoseaua Panduri, 050663 Bucharest, Romania; mihaela.buleandra@g.unibuc.ro (M.B.); irinel.badea@chimie.unibuc.ro (I.A.B.)
7 Research Institute of the University of Bucharest, 050095 Bucharest, Romania; gratiela.gradisteanu@icub.unibuc.ro
8 Department of Inorganic Chemistry, Physical Chemistry and Electrochemistry, Faculty of Chemical Engineering and Biotechnologies, University Politehnica of Bucharest, 1–7 Gh. Polizu Street, 011061 Bucharest, Romania
9 Academy of Romanian Scientists, 3 Ilfov Street, 050045 Bucharest, Romania
* Correspondence: eliza.oprea@g.unibuc.ro (E.O.); anton.ficai@upb.ro (A.F.)

**Citation:** Ilie, C.-I.; Oprea, E.; Geana, E.-I.; Spoiala, A.; Buleandra, M.; Gradisteanu Pircalabioru, G.; Badea, I.A.; Ficai, D.; Andronescu, E.; Ficai, A.; et al. Bee Pollen Extracts: Chemical Composition, Antioxidant Properties and Effect on the Growth of Selected Probiotic and Pathogenic Bacteria. *Antioxidants* **2022**, *11*, 959. https://doi.org/10.3390/antiox11050959

Academic Editors: Evangelos Zoidis

Received: 8 April 2022
Accepted: 10 May 2022
Published: 12 May 2022

**Publisher's Note:** MDPI stays neutral with regard to jurisdictional claims in published maps and institutional affiliations.

**Copyright:** © 2022 by the authors. Licensee MDPI, Basel, Switzerland. This article is an open access article distributed under the terms and conditions of the Creative Commons Attribution (CC BY) license (https://creativecommons.org/licenses/by/4.0/).

**Abstract:** This paper evaluated the chemical and biological properties of bee pollen samples from Romania. Firstly, the bee pollen alcoholic extracts (BPEs) were obtained from raw bee pollen harvested by *Apis mellifera carpatica* bees. The chemical composition of BPE was obtained by determination of total phenol content and total flavonoid content, UHPLC-DAD-ESI/MS analysis of phenolic compounds, and GC-MS analysis of fatty acids, esters, and terpenes. Additionally, the antioxidant activity was evaluated by the Trolox Equivalent Antioxidant Capacity method. Furthermore, the biological properties of BPE were evaluated (antimicrobial and cytotoxic activity). The raw BP samples studied in this paper had significant phenolic acid and flavonoid content, and moderate fatty acid, ester, and terpene content. P1, P2, and P4 have the highest TPC and TFC levels, and the best antioxidant activity. All BPEs studied had antimicrobial activity on pathogenic strains isolated from the clinic or standard strains. A synergistic antimicrobial effect of the BPEs was observed along with the soluble compounds of *L. rhamnosus* MF9 and *E. faecalis* 2M17 against some pathogenic (clinical) strains and, considering the tumour proliferation inhibitory activity, makes BP a potential prebiotic and antitumour agent for the gut environment.

**Keywords:** bee pollen; phenolic compounds; antimicrobial activity; prebiotics; tumour proliferation inhibitory agents

Supplementary material

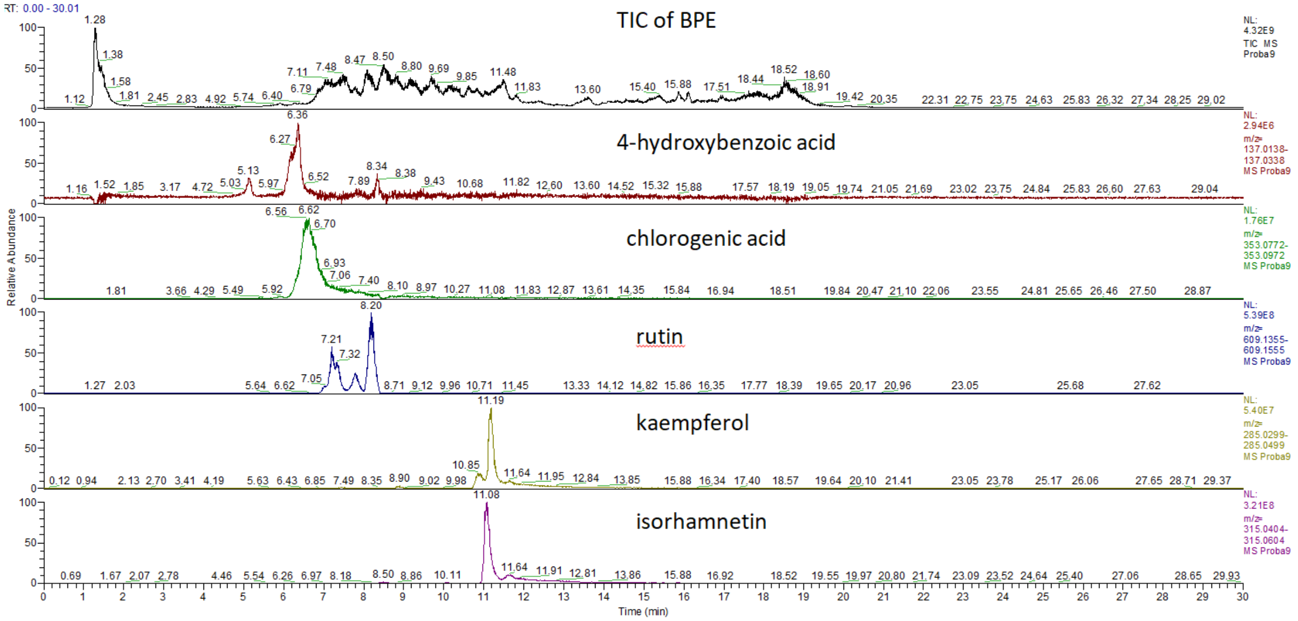


**Figure S1.** TIC and the extracted chromatograms of the main phenolic compounds identified in BPE (the chromatogram were extracted from TIC using a 5 ppm mass accuracy window; negative ion mode, full scan, mass range 100-1000 m/z).
